# Forest Attendance in the Times of COVID-19—A Case Study on the Example of the Czech Republic

**DOI:** 10.3390/ijerph19052529

**Published:** 2022-02-22

**Authors:** Vilém Jarský, Petra Palátová, Marcel Riedl, Daniel Zahradník, Radek Rinn, Miroslava Hochmalová

**Affiliations:** Faculty of Forestry and Wood Sciences, Czech University of Life Sciences Prague, Kamýcká 129, 16500 Praha, Czech Republic; palatovap@fld.czu.cz (P.P.); riedl@fld.czu.cz (M.R.); zahradnik@fld.czu.cz (D.Z.); rinn@fld.czu.cz (R.R.); sodkova@fld.czu.cz (M.H.)

**Keywords:** COVID-19 pandemic, forest attendance, government restrictions, Czech Republic, payments for ecosystem services

## Abstract

The COVID-19 pandemic affected people all over the world, including the Czech Republic (CZ). In the CZ, a number of measures were applied in 2020 to reduce the contact between people and their mobility. This article dealt with the importance of forests during the pandemic. Data from 2019 and 2020 were compared. The qualitative data were obtained from two nationwide surveys, the first focused on forest attendance and forest fruit collection (about 1000 respondents per year), the second on the motivation to visit the forests (about 3700 respondents per year). The quantitative data were obtained on the regional level by analysing data from mobility counters. The impact of government restrictions was assessed. Findings: (1) there was a significant increase in the number of people who frequently visited the forest in 2020; (2) in 2020, the amount of households that collected forest fruits increased and was the highest for the monitored period; (3) the increased forest attendance significantly corresponded to the government restrictions. The analysis confirmed the great importance of forests for the citizens and, at the same time, the increased pressure on the forests’ use—forest attendance and forest crops picking—(especially suburban ones) in times of COVID-19 restrictions.

## 1. Introduction

The current SARS-CoV-2 pandemic, which first appeared in China in 2019, is a global problem that has also affected the Czech Republic (CZ). The first proven infection in the CZ dates back to 1 March 2020, while the negative development of the pandemic situation continued throughout 2020 and in the first half of 2021. Up to 1 July 2021 in the Czech Republic, 1,667,807 people were identified as having contracted COVID-19, while the death toll exceeded 30,000 people [1].

The development of the disease and the burden/overload of the health-care system was followed by a number of anti-epidemic measures to prevent the further spread of the disease in the Czech Republic, especially in the form of restrictions that led to the reduced mobility of the population (e.g., closing shops, schools, the possibility to work from home, etc.). Many of these measures were linked to the so-called state of emergency. The first state of emergency was declared on 12 March 2020 for a period of 30 days and was gradually extended until 17 May 2020. As part of other measures, another state of emergency lasted from 5 October 2020 to 11 April 2021 (with exceptions, such as travelling to work, to a doctor, etc.), for more details, see the [2]. Even after the end of the state of emergency, some restrictions on the movement and gathering of people persisted. These limitations were accompanied by the notion of a lockdown, which led to changes in the normal behaviour of the general population and their leisure time, such as a decrease in their physical activities [3,4,5], including that of children [6]. The coronavirus pandemic has had a major impact on the mobility of the population, including within the local and regional authorities. However, the national mobility of the population is a typical phenomenon in the Czech Republic, especially in connection with commuting to work. A typical example is the connection between the capital city of Prague and the Central Bohemian Region, with respect to the localities Prague-East and Prague-West, which are immediately adjacent to the capital. People frequently commute to work in Prague from the surrounding areas during the workweek. Vice versa, after work, the inhabitants of the surrounding areas commute back to their place of residence, while those who reside in Prague drive back and forth within the framework of leisure activities and spending time in nature [7].

Although it is difficult to predict all the effects of COVID-19 on the population, there is a general agreement that the COVID-19 pandemic affects not only one’s physical health, but also one’s mental health [8,9]. As early as March 2020, the WHO drew attention to the need to consider the effects of a pandemic on human mental health [10]. The consequences of a coronavirus pandemic and related government restrictions include stress, anxiety, depression, frustration, and uncertainty [11,12]. A study was conducted in China to confirm that anxiety, depression, alcoholism, and poorer well-being occurred during the COVID-19 pandemic. The results also showed that people aged 21–40 were more vulnerable with regard to their mental health and alcohol use [13]. Social stigmas and xenophobia are also associated with the COVID-19 situation [14]. The long-term impact of isolation/quarantine on a person’s dysfunctional mental state is also a problem [15]. A study carried out in the Czech Republic by the National Institute of Mental Health identified an increased incidence of mental illness in the adult population, a three-fold risk of suicide and depression, and an increase in the frequency of binge drinking [16].

Various leisure activities, sports, and meeting family and friends are typical ways that people compensate for everyday stresses. However, these activities were significantly affected during the pandemic. Even still, a visit to nature was made possible (although while respecting the set rules, especially social distancing) during the times of the strictest restrictions. In the Czech Republic, visiting the forest is one of the most popular leisure activities [17,18,19,20,21,22]. As in Sweden, for example, where the recreational function of a forest is one of the five most important ecosystem services [23]. The proximity of the forest has positive psychological and physical effects for the population [24]. The positive effects were mentioned by Karjalainen et al. [25], specific positive effects on mental health include, for example, the reduction of stress, depression, and burnout, and beneficial effects on the cardiovascular and immune system [26]. The effects of visiting an urban forest and city park were investigated by the study of Hansmann et al. [27], where the respondents rated their headaches and level of stress in connection to visits to an urban forest and city park in Zurich, Switzerland.

Recently, a number of studies analysing the importance of the forest during this COVID-19 period have appeared. The effects of the coronavirus pandemic on forest attendance were, for example, part of a study by Derks et al. [28]. In Germany, in the Kottentforst area (located west of Bonn), the attendance has increased, especially in the afternoon and on weekdays, as a likely consequence of the lockdown situation. Venter et al. [29] dealt with the situation in Oslo, Norway, where it was also possible to spend time “outside” during lockdowns. The outdoor recreational activities increased there by 291% in 2020 compared to the 3-year average, from 28,000 to 114,000. In Vermont, USA, they also confirmed increased attendance at urban forests and parks in the Burlington area [30]. The study also focused on the reasons for visiting this site, the main ones being “just getting outside”, “exercise”, and “connecting to nature”. “Almost 40% of respondents reported that they utilised visiting natural areas in connection with activities that reduce stress and rumination”. The increase in forest attendance as a result of the coronavirus situation was also confirmed by the results of a study carried out in Slovakia [31].

However, the results published so far were mainly based on “fast” research methods, mainly surveys among forest visitors. More complex analyses are still lacking. In general, detecting and measuring forest attendance is possible on site, by querying, or using modern methods (GPS, eco-counters, etc.). In addition, the information obtained regarding forest attendance is also important for forest management, in relation to the issue of forest ecosystem services and in relation to the forest’s owner [32].

The purpose of the presented analysis was to provide a more comprehensive idea of the impact of the COVID-19 pandemic on forest attendance in the Czech Republic. This was undertaken by using a number of methodological procedures examining changes in the long-term trends in forest attendance and forest fruit collection and supplementing them with a case study of forest attendance in a selected region with the help of automated counters (eco-counters). The aim was to answer the following research questions:

RQ1: Did the restrictions related to the COVID-19 pandemic increase forest attendance and motivation to visit the forest?

RQ2: Has there been a change in the intensity of forest fruit harvesting due to restrictions related to the COVID-19 pandemic?

RQ3: Is it possible to identify other factors at the local level that significantly affected forest attendance in 2020?

## 2. Materials and Methods

Methodologically, the research was divided into two levels: national and local.

### 2.1. National Level

This analysis was based on the use of modern statistical methods in processing and comparing the results of 2020 in two parallel surveys at the national level with previous years, which dealt with (1) the frequency and the intensity of the forest fruit harvest and (2) the motivation of the forest visits. Particularly speaking about the following:

#### 2.1.1. Analysis of the Changes in the Forest Visits and in the Non-Wood Forest Products (NWFP) Picking

Since the year 1994, standardised questions related to forest visits and the amount of harvested wild berries and mushrooms per household were used in personal interviews. For details on the methodology and results, see Sisak et al. [21] for mushrooms and Riedl et al. [19] for forest fruits. For the period of 1994–2007, only the aggregated data estimating the total amount and the total value of the collected forest berries are available; for the period 2008–2020, the socio-economic data of the individual respondents including the amount and price per kg of the collected forest berries are available. Since 2008, the survey has been conducted by the hired research agency Stemmark, who, as a member of the European association of research agencies (ESOMAR), meets the strict criteria regarding ethics and a professional approach to market research. The questionnaire was conducted using a computer-aided personal interview method (CAPI) on a representative group of respondents as a part of the omnibus research. The network of approximately 250 trained interviewers allowed one to achieve the desired representativeness of the research sample using the original standardised questionnaire. The respondents were selected on the basis of their gender, age, education, size of the municipality, and region of residence (a so-called quota sample) which ensures the representativeness of the sample with respect to these criteria, and which is also an important prerequisite for the subsequent statistical analyses. Between 2008 and 2020, the number of respondents in the annual survey ranged between 1000 (2013) and 1087 (2011). There were 1075 respondents in the year 2019 and 1001 in 2020. The investigation in the year 2020 took place between 20.11.–5.12. 2020, so the results take almost the whole of 2020 into account.

#### 2.1.2. Analysis of the Changes in the Motivation of Forest Visits in the Czech Republic

Data on the sociological research mapping the frequency and reasons for visits to the forest by the general public has been ongoing as part of the Market & Media & Lifestyle (MML-TGI) Research Project. That investigation has been carried out in licensed cooperation with Kantar Media UK Ltd., Prague. 

For details on the research methodology, see Šodková et al. [22]. Of the 8.8 million inhabitants of the Czech Republic aged 12–79 years old, the respondents were selected by using the quota selection method including the following criteria: gender, age, level of education, monthly income, and size of residence. The number of recruited representative respondents in the research project was 3750 in 2019 and 3733 in 2020. The questioning was conducted by the method of face-to-face interviews with an interviewer in combination with using the proven computer-assisted web interviewing (CAWI) method. The MML-TGI project contains four main spheres of questions: a personal data part (face-to-face or online method), including sociodemographic data; a media part (face-to-face or online method); a consumer behaviour part (prevailingly undertaken by an independent fill-out form); and a lifestyle part (prevailingly undertaken by an independent fill-out form) including 620 entries on the respondent’s lifestyle. From this wide collection of questions, those related to forest attendance and fruit collection were used for our research. Using this methodological procedure, we investigated whether the COVID-19 pandemic has changed the motivations to visit forests.

### 2.2. Quantitative Analysis of the Changes in Forest Attendance of a Selected Region–Local Level

**Figure 1:**
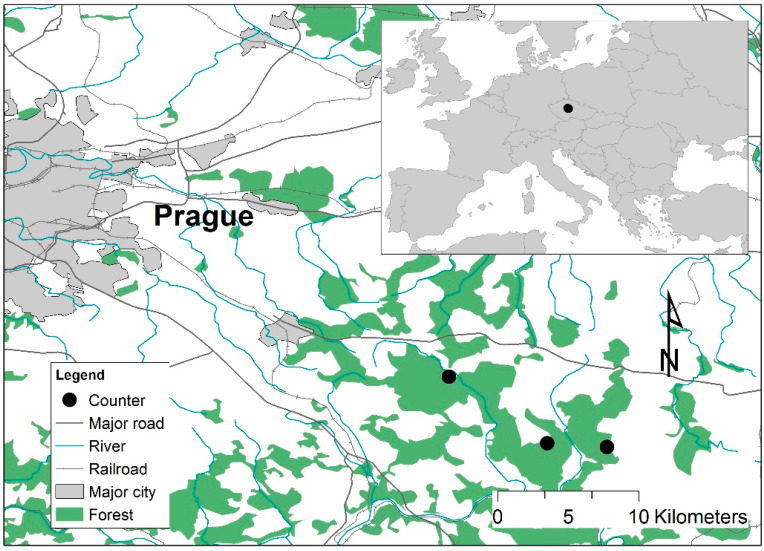
Location of mobility counters (from left: Jevany, Oplany, Kachní louže).

For this quantitative analysis, a locality was chosen in the vicinity of the town of Kostelec nad Černými lesy in the Central Bohemian region, approximately 30 km from the capital city of Prague (hereinafter referred to as Černokostelecko). The investigated locality is located near the Voděradská bučiny National Nature Reserve. This extensive forest complex is characterised by a varied species composition and beech stands close to nature, and there is also a water area suitable for recreation nearby. This makes this location an attractive place to spend free time and use other ecosystem services not only for local residents, but also significantly for the inhabitants of Prague. At this research site, automated mobility counters, Eco-counter Mobil Multi (https://www.eco-counter.com/produits/multi-range/mobile-multi/, accessed on 10 December 2021), were installed, enabling the registration of visitors with a distinction between pedestrians and cyclists, including the direction of passage and transit. Three counters were installed with different distances both from the main tourist centre of the locality (Jevany) and from Prague (see Figure 1).

The analysis was performed by comparing the years 2019 (year without COVID-19) and 2020 (year with COVID-19). As the weather conditions logically affect the number of visitors, data on the local precipitation and temperatures were also included in the analysis. These data were obtained from the publicly accessible records of the Czech Hydrometeorological Institute (CHMI). Data from the meteorological station Ondřejov were used. This station is the closest to the sites where the research was carried out (Jevany—6 km, Oplany—6 km, Kachní louže—10 km). 

The actual impact of COVID-19 in the Czech Republic was reflected in government restrictions, which we divided into five categories: A—closing shops and restaurants, B—closing schools, C—restricting sports activities, D—restricting cultural events, and E—restricting travel (beyond the borders of the Czech Republic). Each day from 2019 and 2020 was assigned with daily data from counters and information on the temperature and precipitation and the validity of a specific restriction.

### 2.3. Statistical Methods

Standard descriptive statistics techniques such as tables and bar charts were used to present the data. Contingency tables were presented graphically using mosaic display. A chi-square goodness-of-fit test was used to evaluate the significance of the difference in attendance across years. Poisson’s regression model was used to evaluate the effect of various factors on forest attendance. Here, average daily temperature, daily total precipitation, and restrictions valid on a given day were used as explanatory variables. The dependent variable was the number of visitors per day at all three sites in total. The statistical analyses were carried out by the R programming environment.

## 3. Results

### 3.1. Long-Term Development of Attendance and Motivation to Visit Forests

**Figure 2:**
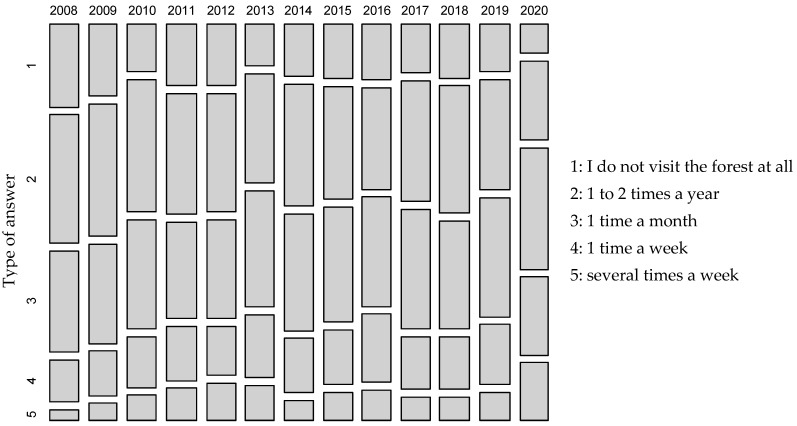
Frequency of forest visits by category; answers to the question: How often do you visit the forest on average per year?

As mentioned in the Material and Methods point Section 2.1.1, respondents were questioned about the intensity of forest visits. Figure 2 shows a comparison of the frequency of the forest visits in the years 2008–2020. A five-point scale of forest visit frequency of 1–5 was chosen, where 1—the forest was not visited at all, 2—forest visits happened very rarely (1–2 times a year), 3—more frequent visits (once a month), 4—quite often (once a week) and 5—very often (several times a week). The Figure 2 shows the shares of each category in a particular year. A mosaic display chart was used for better visual comparison, where a larger box represents a larger proportion (share).

The results showed a clear increase in the attendance in 2020, especially (compared to the last few years), the number of very frequent visitors (category 5) doubled—it was the highest in the entire period under review. On the other hand, the number of people not going to the forest (1) was halved and was the smallest in the observed period. The differences were statistically highly significant (the level of significance of the chi-square test of the homogeneity of multinomic distributions was less than 2.10^−16^). In 2020, 70.5% of the people questioned went to the forest at least once a month, compared to 56.7% in 2019. At the same time, the number of people who did not want or could not visit the forest decreased from 12.8% in 2019 to 7.8% in 2020. The attendance in the monitored sample of respondents was the highest in absolute numbers for the entire monitored period.

**Figure 3:**
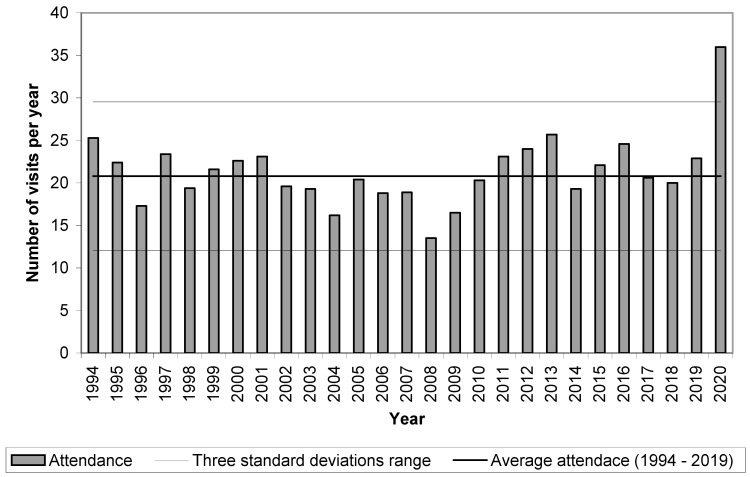
Attendance per inhabitant of the forest accessible to the public in the Czech Republic in the period of 1994–2020.

Based on the data analysed according to Section 2.1.1., Figure 3 illustrates the specificity of the year 2020. The annual attendance per inhabitant in 2020 clearly exceeded the three standard deviations range in 1994–2019. Similarly, we could evaluate an annual attendance per hectare. The population and forest area in the Czech Republic evolved differently between 1994 and 2020, but the changes were two orders of magnitude smaller than the values of these variables alone. The evolution of attendance per hectare was therefore almost identical to the evolution of attendance per inhabitant.

The research on forest visits according to Section 2.1.2 as mentioned in the Materials and Methods section did not examine forest attendance as a whole but examined the changes in the answers to the question related to the number of forest visits for specific reasons. The division was made into three reasons for visiting the forest: a walk (the joy of staying in nature), sports, and picking berries.

Table 1 confirms that there was an increase in the share of more frequent forest visits and a decrease in the share of sporadic visits in 2020, for all types of motives for visiting the forest. The differences were highly significant, and the chi-square test rejected the hypothesis of same distribution in years 2019 and 2020 at *p*-values 2.2 × 10^−16^, 1.3 × 10^−12^, and 8.9 × 10^−14^ for the listed reasons for visiting.

### 3.2. Changes in the Forest Fruits Collection

In the Czech Republic, the intensity of the forest fruit harvest has long been monitored for the six most common items. The most important ones are the collection of mushrooms and blueberries. The results were obtained by analysing the data obtained according to Section 2.1.1.

It is clear from Table 2 that the number of households collecting forest crops increased for all types of monitored forest fruits, e.g., mushrooms were harvested in 2020 by over 80% of Czech households, blueberries by almost two-thirds, and raspberries and blackberries by about half of the households. To demonstrate the specificity of 2020, Figure 4 shows a year-on-year comparison of the five previous years, from 2015 to 2019.

**Figure 4:**
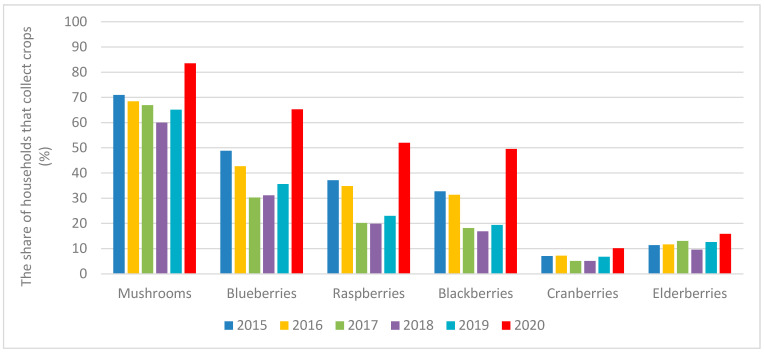
Collection of wild berries; answers to the question: What fruits do you pick in the forest?

Compared to 2019 and 2018, the number of households that collected mushrooms and berries increased. From Table 2 and Figure 4, it is evident that although the number of households collecting the forest fruits increased for all the monitored commodities, the most frequently collected commodities, such as mushrooms and blueberries, raspberries and blackberries, decreased in relation to the volume of the collection attributable to the collecting household. The share of households that collect mushrooms and berries in 2020 increased significantly not only compared to 2019, but even reached historically high values.

### 3.3. Changes in the Number of Visitors to the Černokostelecko Locality

**Figure 5:**
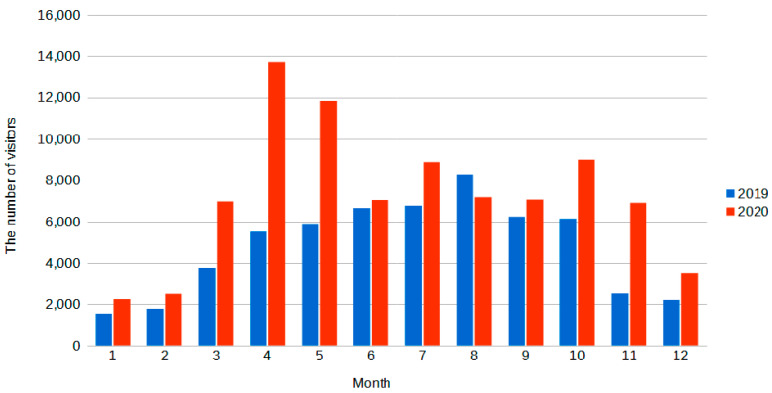
Number of visitors to the locality in the individual months of 2019 and 2020.

The census data (based on the data analysed according to Section 2.2.) showed that, in 2019, 57,236 people visited the examined sections, while, in 2020, 86,742 people visited the same locations: an increase of 52%. As Figure 5 shows, the increase is particularly noticeable in the months of March–May, i.e., during the period of validity of the largest restrictions, and subsequently in the autumn months, when further restrictions were introduced in connection with the second wave of the disease. A summary of the data for all three counters is displayed, regardless of whether they were pedestrians or cyclists.

Of the five restriction areas resulting from the government restrictions, the closure of shops and restaurants (restriction A) was considered the most important. The following information in Table 3 shows how statistically significant this restriction was, even compared to the weather.

The results for the other restrictive measures were very similar.

Poisson’s regression model [33], applied for the analysis in Table 3, clearly documents the effect of all the variables. Namely, the correlation between the attendance rate and the temperature was positive (see the estimate-relevant coefficient) and statistically highly significant (*p* value < 2 × 10^−16^). As expected, the effect of precipitation on the attendance was negative and statistically highly significant. The effect of closing the shops and restaurants was positive, i.e., a statistically significant higher attendance of the forests was recorded on the days this restriction was valid.

More detailed information is provided in Table 4, in which all types of government restrictions are assessed in the same way, in addition to the division into pedestrians and cyclists.

As Table 4 shows, in all the models, the significance levels for all the variables were less than 2 × 10^−16^, so they were very highly conclusive. The values of the test statistics (z) suggested that the most important factor for pedestrians was the announced restrictions, while for cyclists, it was the daily temperatures.

Comparing the influence of the individual types of restrictions was problematic. All kinds of restrictions were applied in very similar periods, which was evident from the results, which were very balanced. The given values only indicated that closed restaurants and shops had the greatest influence for pedestrians, and closed schools, for cyclists.

The detailed analysis for the individual localities also indicated a slight decrease in the effect of the restrictions on the attendance with the increasing distance of the locality from Prague.

## 4. Discussion

The data presented summarised the situation of forest attendance (and related forest fruit harvesting) in 2020 and compared it with 2019. The purpose of the research was not to create a synthesis of results related to the different datasets used (and the reasons behind it), but to show that it is possible to confirm the importance of COVID-19 (respectively, anti-COVID-19 measures) based on different research. It should be emphasised that no survey was directly or indirectly related to COVID-19 (i.e., whether the respondents visited the forest due to the government restrictions related to the pandemic). Such a focus of questions could lead to a strategic action by the respondents [34]. The impact of the pandemic was, thus, analysed indirectly, and the fact that COVID-19 (or related government restrictions) was a major reason for the increasing forest attendance and forest fruit collection was a logical result of the analysis.

Our analysed data are from two research studies. One research studied the frequency of forest visits and the amount of forest fruit collected. The other research studied different motives for forest visits. Because the data were from independent studies, the connection of the amount of forest fruits collected and the main motive of forest visits could not be statistically confirmed. 

Forests, apart from their multifunctional role, also underpin the important sectors such as public health, employment, and disaster risk reduction [35]. The results of the increased forest attendance during the coronavirus pandemic confirm the irreplaceable role of forests and emphasise the importance of the ecosystem services they provide, especially in suburban areas. The increased attendance of urban forests in the Czech Republic (locality of Hradec Králové) was analysed by Jůza at al. [32]. A study by Weinbrenner et al. [36] confirmed the critical importance of urban forests during lockdowns. The increase in attendance in selected forests in connection with the coronavirus pandemic was confirmed in European localities by [28,29,32]. Wunder et al. [37] also pointed to a change in the behaviour of visitors, when not only the time of forest visits during the day changed, but also the presence of visitors who did not visit forests at all before the COVID-19 pandemic (especially young people, people with a place of residence outside the monitored locality). This is, probably, comparable to our results, which show that the group of people who did not visit the forest at all in 2020 has significantly decreased. Derks et al. [28] stated that occasional visitors, however, are not aware of how to behave in the forest. Therefore, it is necessary to increase not only their awareness, but also to create conditions for the implementation of the necessary forestry activities in the forest, and, at the same time, to be accessible to visitors—we recommend performing work in the forest early in the morning, because it is around this time when there are few visitors.

The increase in the number of visitors to natural sites has also been observed in national parks [38], which confirms the increased interest in visiting these areas during the pandemic. However, this increase also caused new problems that appeared in the given localities (overcrowding, irresponsible users (waste, littering, noise), parking and traffic issues, conflicts between local people and visitors, etc.). So, nature locations must also adopt measures that ensure that a certain locality will be able to absorb the visitors, but with either no or minimal negative impacts on the nature area. We also make some recommendations for the management of nature parks, such as careful spatial planning, closer collaboration between park authorities and more centralised institutions, and to avoid over-tourism.

It is the congestion of sites caused by increased attendance that may jeopardise the sustainable provision of ecosystem services in the future and may cause additional pressure not only on the forest management, but also on the local site. Therefore, it is important to monitor these potential impacts and initiate a discussion between the local authorities and forest owners, with respect to state administration bodies.

One of the tools that can mitigate the effects of over-use of some ecosystem services is the so-called payments for ecosystem services (PES). PES can be defined as voluntary transactions between the service(s) users and service providers that are conditional on agreed rules of natural resource management for generating offsite services [39]. For example, a pilot project for payments for ecosystem services was initiated in Costa Rica in 1997 with the aim of maintaining and improving the provision of environmental services in forestry [40]. The climate, water sources, biodiversity, and clean air are the environmental inputs of all the products and services. However, their value is still not sufficiently economically taken into account and quantified. Their quantification is necessary to determine compensation for the use of the services and, thus, to achieve sustainable forest use [41,42,43]. For example, a study by Davies et al. [44] found that private companies are willing to volunteer to support ecosystem services provided by urban forests and green spaces in the vicinity, with the aim of supporting their business and strengthening their marketing position in the market. One of the ways to reduce the excessive collection of forest fruits (especially mushrooms) could be, for example, to change one of the forms of property rights (withdrawal and exclusion rights), so that the forest fruits belong to the landowner (so he could decide on further disposal). Various analyses have been carried out, especially in the Mediterranean area, that show [45,46,47] that tools can then be used that enable the public to pick forest fruits and, at the same time, increase the income of the forest owner.

The production, and especially the wood production function of forests, is still preferred within the benefits provided in the CZ due to the economic benefits it brings to the forest owner. However, its importance may decrease in the future, for example, in connection with the bark beetle calamity and climate change, to which the species composition of the forests must be adapted. Support for a forest’s multifunctional role will, thus, be necessary and will need to be taken into account in decision-making processes concerning forest management. Entry into the forest is free in the Czech Republic, with a few exceptions [48], with respect to the owner’s point of view, visitors to the forest, including the activities they carry out in the forest (i.e., also the collection of forest crops) have become something that the forest owner has to endure and that can reduce the owner’s economic benefit. As the above-mentioned analyses show, the non-wood production function of forests is also of growing importance in the CZ. This was confirmed by a recent analysis of NWFP in European countries by Lovrić et al. [49].

It is clear from the above that any recommendations for forest management must take a large number of factors into account, including a thorough analysis of the forest visitors. As reported by Kajala et al. [50], monitoring the attendance is an important factor in supporting management and outdoor tourism. The problem is often conflicting interest and the impact of the needs of the individual stakeholders on the forest, which may be reflected in different recommendations for the forest management depending on who (and for whom) they are created [51,52,53].

## 5. Conclusions

The results of the submitted analyses clearly show that the COVID-19 pandemic led the inhabitants of the Czech Republic to make a significant change in their forest attendance. This was confirmed by research on both national and regional levels.

It is, therefore, possible to clearly answer YES to RQ1. (Have the restrictions related to the COVID-19 pandemic increased forest attendance and motivation to visit the forest?) There was a significant increase in the number of people who frequently went to the forest in 2020, and the number of people who did not go to the forest at all diminished.

As for RQ2 (has there been a change in the forest fruit harvesting intensity due to the restrictions related to the COVID-19 pandemic?), the answer is also YES. In the year 2020, the share of households that collected forest fruits increased and was even the highest during the entire period under review.

As for RQ3 (can other factors be identified at the local level that significantly affected forest attendance in 2020?), can also be answered with YES, as the regional view revealed that increased forest attendance significantly corresponded to the government restrictions, with no significant difference in the pedestrian attendance and cyclists. In addition, the temperature and precipitation at the site logically had an impact on the attendance.

On the one hand, it is clear that access to forests brings benefits to human health at a time when it is most needed. On the other hand, in recent years, many people have reservations about the forest management and believe that there are natural losses in these areas. Forest owners, thus, have the opportunity to convince the public otherwise. The indispensability of forest ecosystem services (especially with an emphasis on suburban forests) appears to be a key stimulus for the development of economic instruments to compensate forest owners for the use of their property, both in the private and public sectors. Assigning value to forest ecosystem services and taking them into account economically is the still an unfulfilled challenge of this millennium.

## Figures and Tables

**Table 1:** Changes in the frequency of forest attendance according to the reason for the visit.

Reason for Visit	Frequency	2019	2020
Just for a walk/for the joy of being in nature	For most of the year-several times per week	12.89%	15.19%
	During holidays (holidays/weekends/holidays)-several times per week	18.44%	23.86%
	Several times per month	22.04%	24.30%
	About once per month	19.22%	16.84%
	Less often (1–4 times per year)	27.42%	19.81%
I play sports in the woods (I run/ride a bike…)	For most of the year-several times per week	12.22%	10.20%
	During holidays (holidays/weekends/holidays)-several times per week	14.44%	18.62%
	Several times per month	19.36%	22.56%
	About once per month	20.44%	15.45%
	Less often (1–4 times per year)	33.54%	33.17%
Collect mushrooms/wild berries/medicinal herbs, etc…	For most of the year-several times per week	1.61%	4.48%
	During holidays (holidays/weekends/holidays)-several times per week	16.08%	16.64%
	Several times per month	11.75%	12.21%
	About once per month	15.35%	17.21%
	Less often (1–4 times per year)	55.21%	49.45%

**Table 2:** Volume of forest crops harvested in kg per household in 2020.

Year	Item	Mushrooms	Bilberries	Raspberries	Blackberries	Cowberries	Elderberries
2019	% of collecting households	65.12%	35.63%	22.98%	19.35%	6.70%	12.56%
	Average per collecting household (in kg)	8.20	3.74	1.53	1.57	1.05	2.85
2020	% of collecting households	83.52%	65.23%	51.95%	49.55%	10.09%	15.78%
	Average per collecting household (in kg)	7.04	2.96	1.60	1.56	1.27	3.14

**Table 3:** Influence of the weather and individual reasons (the restrictions) on the year-on-year number of visitors to the site.

Coefficients	Estimate	Z	*p*-Value
(Intercept)	4.380	671.3	<2 × 10^−16^
Temperature	0.068	170.3	<2 × 10^−16^
Precipitation	−0.029	−39.15	<2 × 10^−16^
Restriction A	0.902	138.47	<2 × 10^−16^
(Intercept)	4.406	696.34	<2 × 10^−16^
Temperature	0.063	162.27	<2 × 10^−16^
Precipitation	−0.033	−44.14	<2 × 10^−16^
Restriction B	0.510	151.18	<2 × 10^−16^
(Intercept)	4.404	697.0	<2 × 10^−16^
Temperature	0.063	162.1	<2 × 10^−16^
Precipitation	−0.032	−43.4	<2 × 10^−16^
Restriction C	0.544	152.1	<2 × 10^−16^
(Intercept)	4.409	699.4	<2 × 10^−16^
Temperature	0.063	161.9	<2 × 10^−16^
Precipitation	−0.032	−43.0	<2 × 10^−16^
Restriction D	0.539	150.7	<2 × 10^−16^
(Intercept)	4.594	795.9	<2 × 10^−16^
Temperature	0.054	144.5	<2 × 10^−16^
Precipitation	−0.033	−44.6	<2 × 10^−16^
Restriction E	0.424	131.2	<2 × 10^−16^

**Table 4:** Statistical significance of the effect of the temperature, precipitation, and individual restrictions.

	Pedestrians			Cyclists		
Coefficients	Estimate	Z	*p*-Value	Estimate	Z	*p*-Value
(Intercept)	4.063	500.8	<2 × 10^−16^	3.371	355.5	<2 × 10^−16^
Temperature	0.029	51.9	<2 × 10^−16^	0.095	172.9	<2 × 10^−16^
Precipitation	−0.031	−27.6	<2 × 10^−16^	−0.026	−27.3	<2 × 10^−16^
Restriction A	0.509	98.9	<2 × 10^−16^	0.607	123.3	<2 × 10^−16^
(Intercept)	4.070	496.9	<2 × 10^−16^	3.318	338.4	<2 × 10^−16^
Temperature	0.027	49.0	<2 × 10^−16^	0.097	171.8	<2 × 10^−16^
Precipitation	−0.035	−31.0	<2 × 10^−16^	−0.029	−30.4	<2 × 10^−16^
Restriction B	0.456	92.5	<2 × 10^−16^	0.581	124.7	<2 × 10^−16^
(Intercept)	4.055	493.1	<2 × 10^−16^	3.332	342.5	<2 × 10^−16^
Temperature	0.028	49.4	<2 × 10^−16^	0.096	171.2	<2 × 10^−16^
Precipitation	−0.034	−30.1	<2 × 10^−16^	−0.029	−30.3	<2 × 10^−16^
Restriction C	0.499	95.9	<2 × 10^−16^	0.607	122.3	<2 × 10^−16^
(Intercept)	4.061	494.9	<2 × 10^−16^	3.338	343.8	<2 × 10^−16^
Temperature	0.027	49.2	<2 × 10^−16^	0.095	171.2	<2 × 10^−16^
Precipitation	−0.034	−29.9	<2 × 10^−16^	−0.029	−30.1	<2 × 10^−16^
Restriction D	0.490	94.4	<2 × 10^−16^	0.603	121.7	<2 × 10^−16^
(Intercept)	4.258	575.8	<2 × 10^−16^	3.501	386.2	<2 × 10^−16^
Temperature	0.020	36.2	<2 × 10^−16^	0.087	160.8	<2 × 10^−16^
Precipitation	−0.037	−31.6	<2 × 10^−16^	−0.030	−30.6	<2 × 10^−16^
Restriction E	0.322	64.9	<2 × 10^−16^	0.529	122.7	<2 × 10^−16^
Null deviance: 54,509 on 730 df				Null deviance: 78,024 on 730 df		
Residual deviance: 43,864 on 727 df				Residual deviance: 38,540 on 727 df		
